# Supplementary figures and images for: Vaccine models predict rules for updating vaccines against evolving pathogens such as SARS-CoV-2 and influenza in the context of pre-existing immunity
Source: Front Immunol. 2022 Oct 3;13:985478. doi: 10.3389/fimmu.2022.985478 (PMC9574365; doi:10.3389/fimmu.2022.985478)

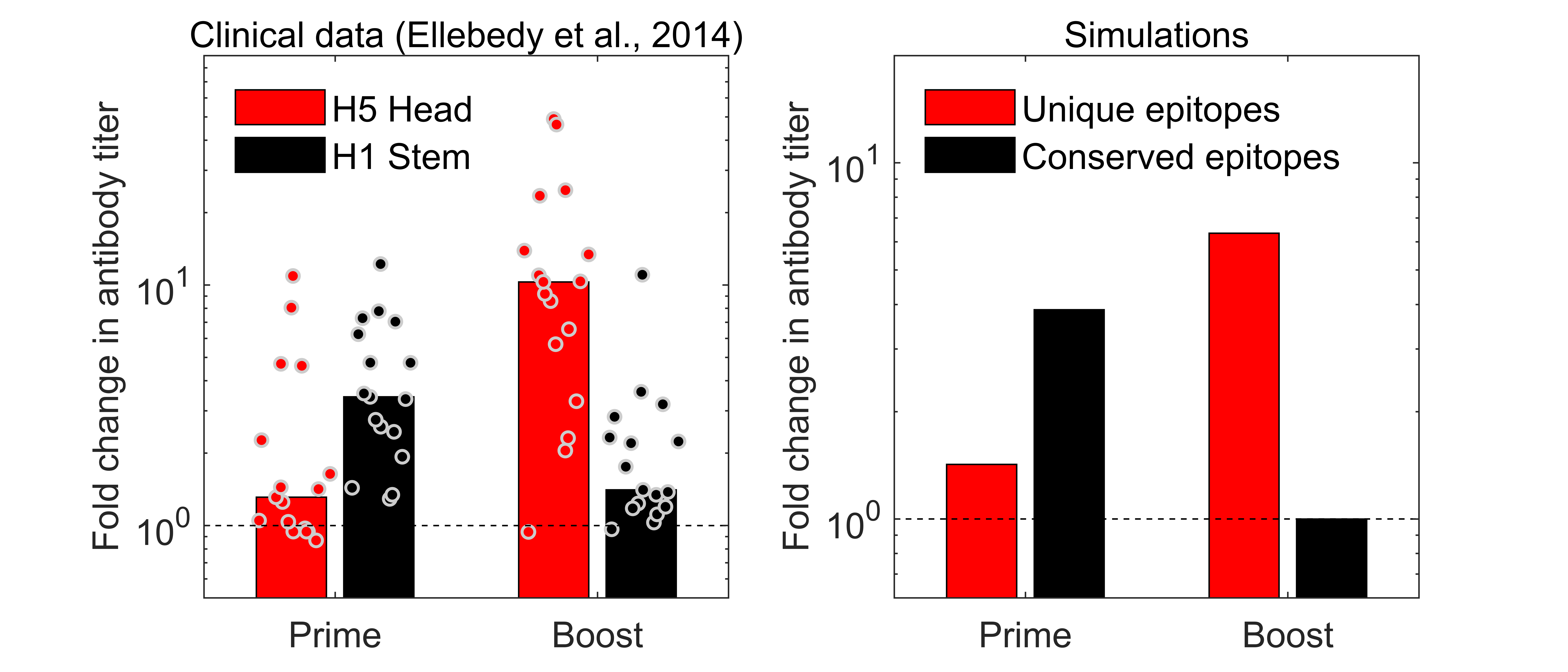

Supplement: Supplementary file 2 [file DataSheet_1.zip › Supplementary_Material_Codes/Fig5.png]

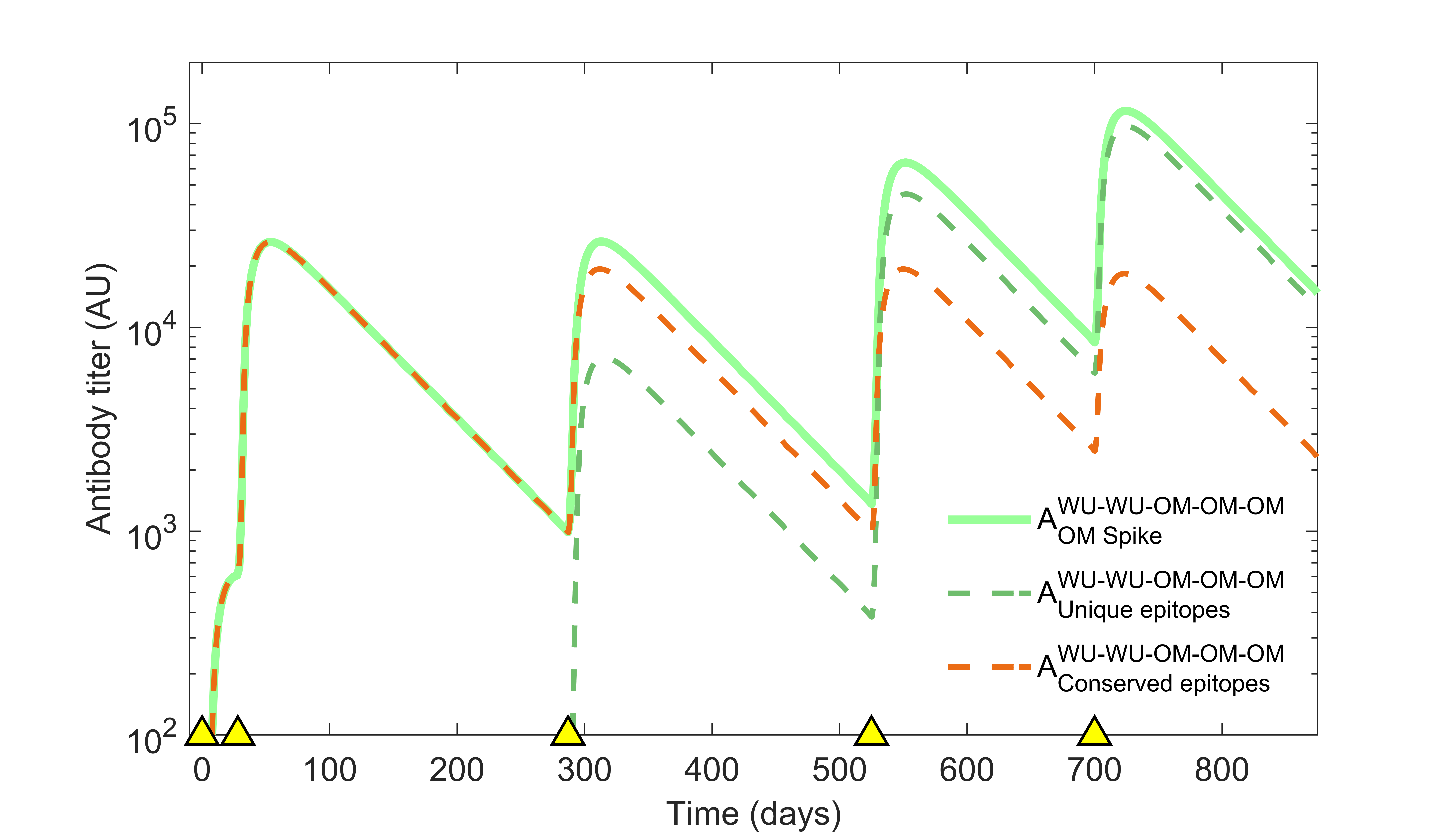

Supplement: Supplementary file 2 [file DataSheet_1.zip › Supplementary_Material_Codes/Fig4B_left.png]

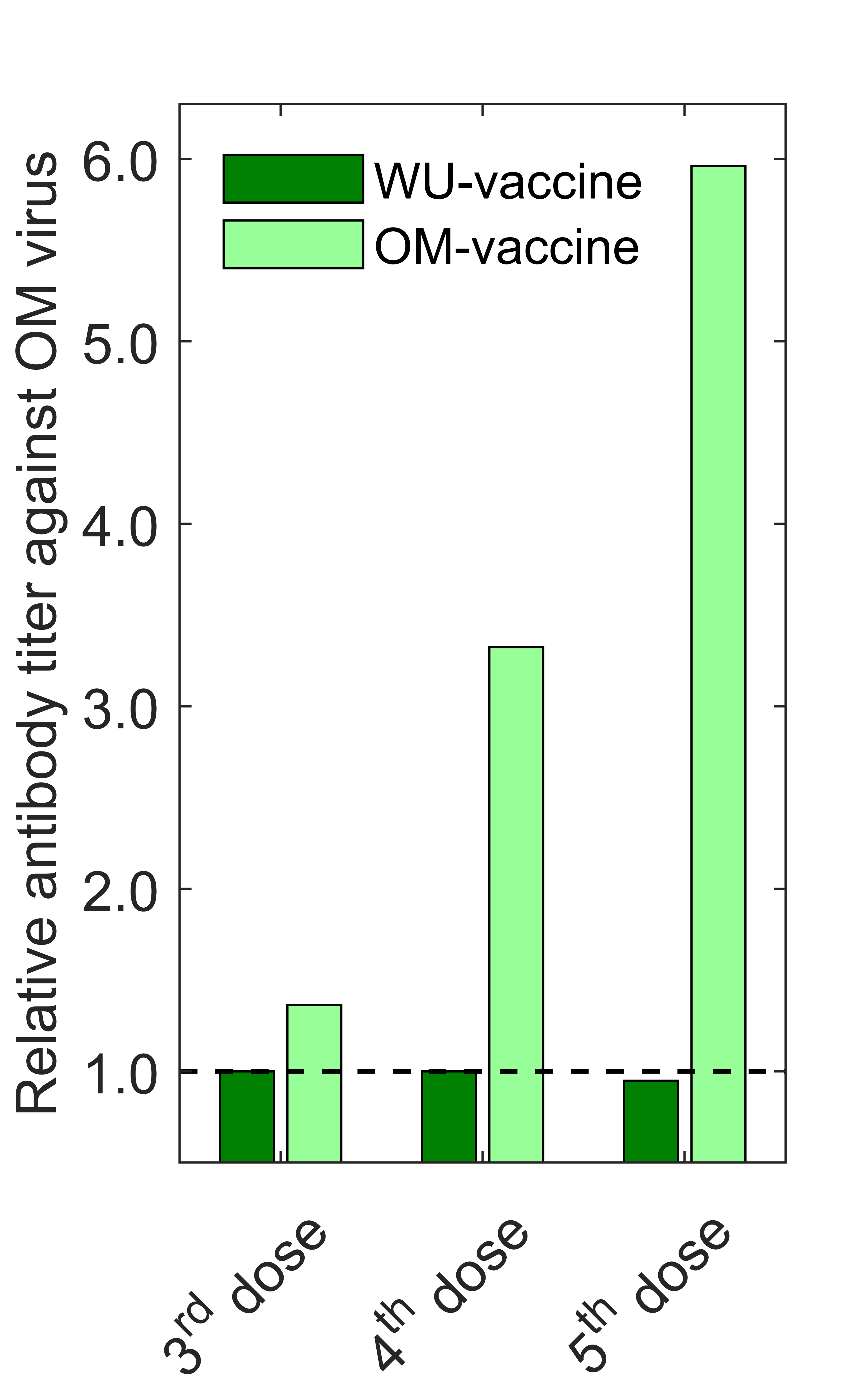

Supplement: Supplementary file 2 [file DataSheet_1.zip › Supplementary_Material_Codes/Fig4A_right.png]

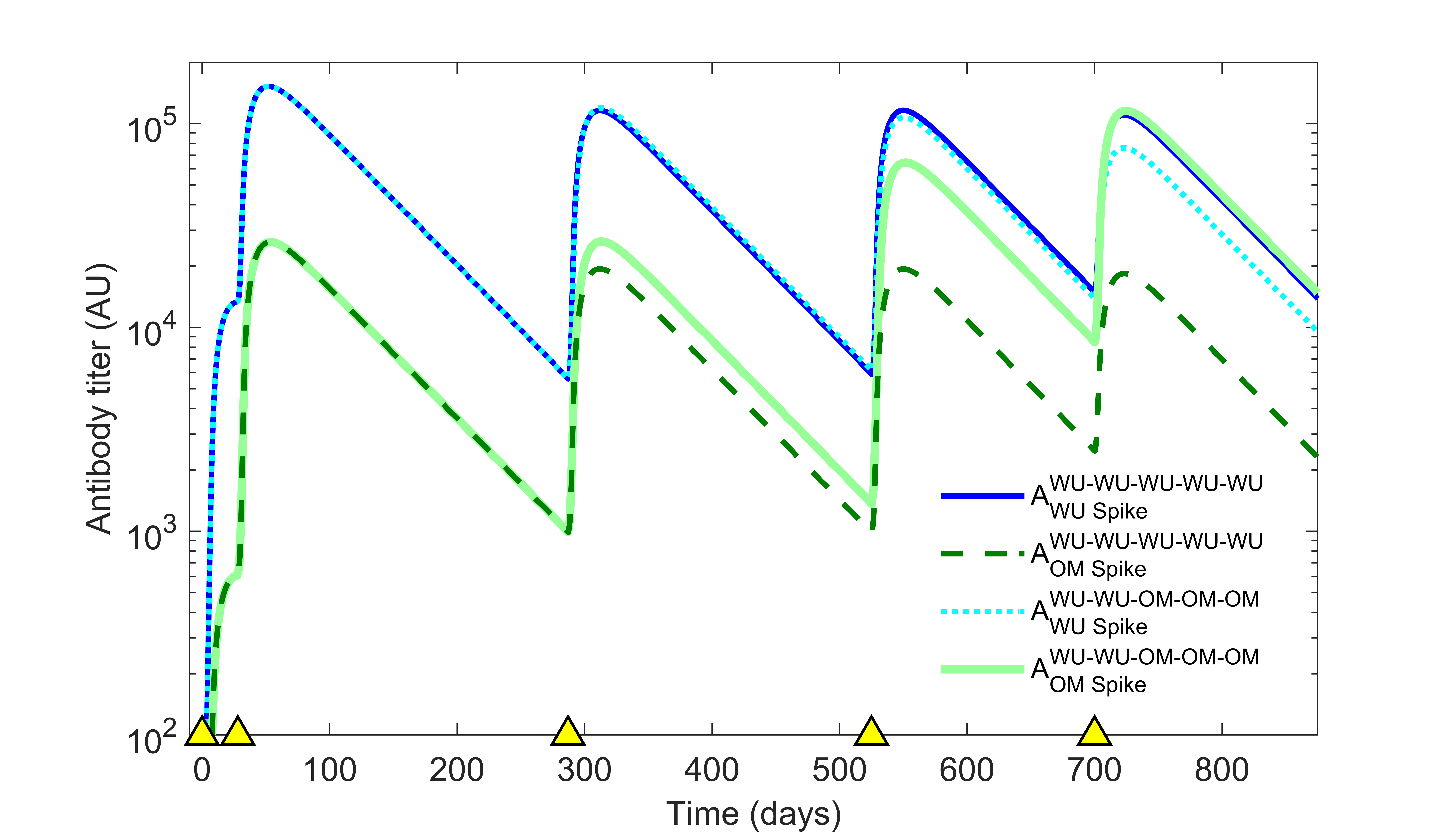

Supplement: Supplementary file 2 [file DataSheet_1.zip › Supplementary_Material_Codes/Fig4A_left.png]

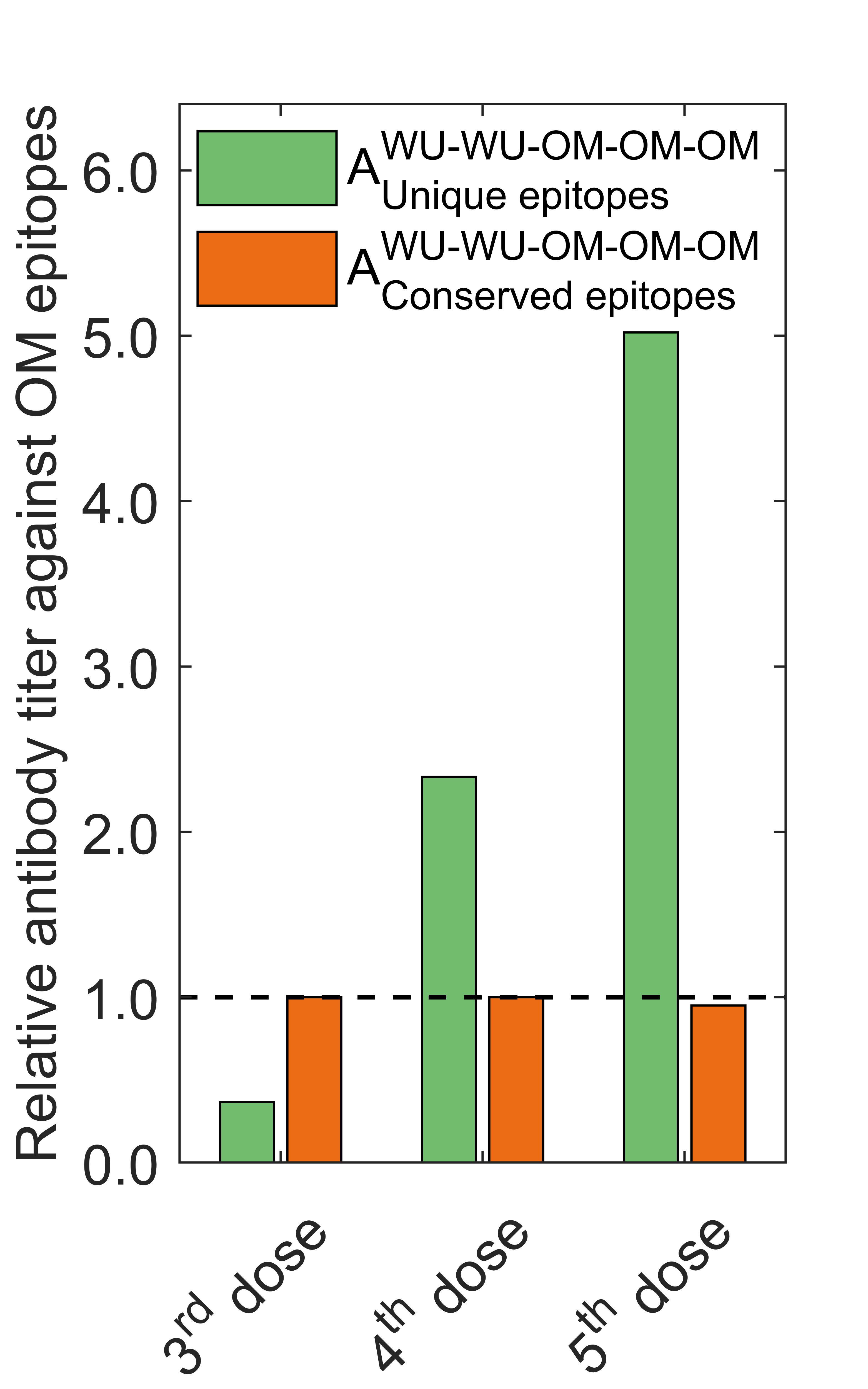

Supplement: Supplementary file 2 [file DataSheet_1.zip › Supplementary_Material_Codes/Fig4B_right.png]

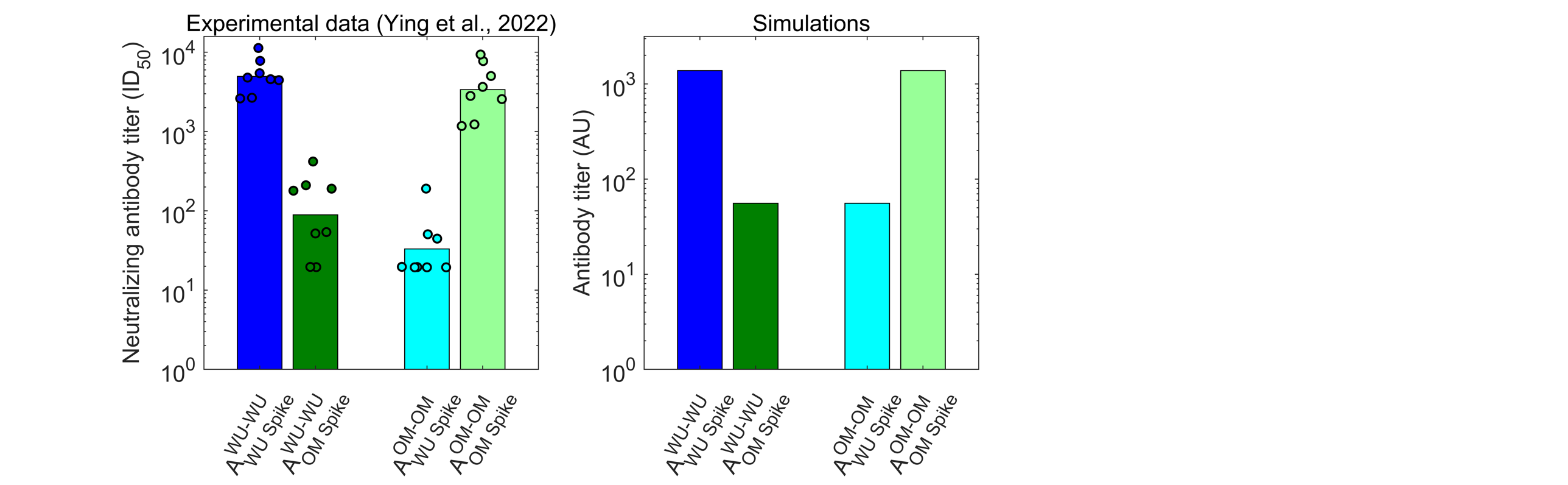

Supplement: Supplementary file 2 [file DataSheet_1.zip › Supplementary_Material_Codes/Fig2A.png]

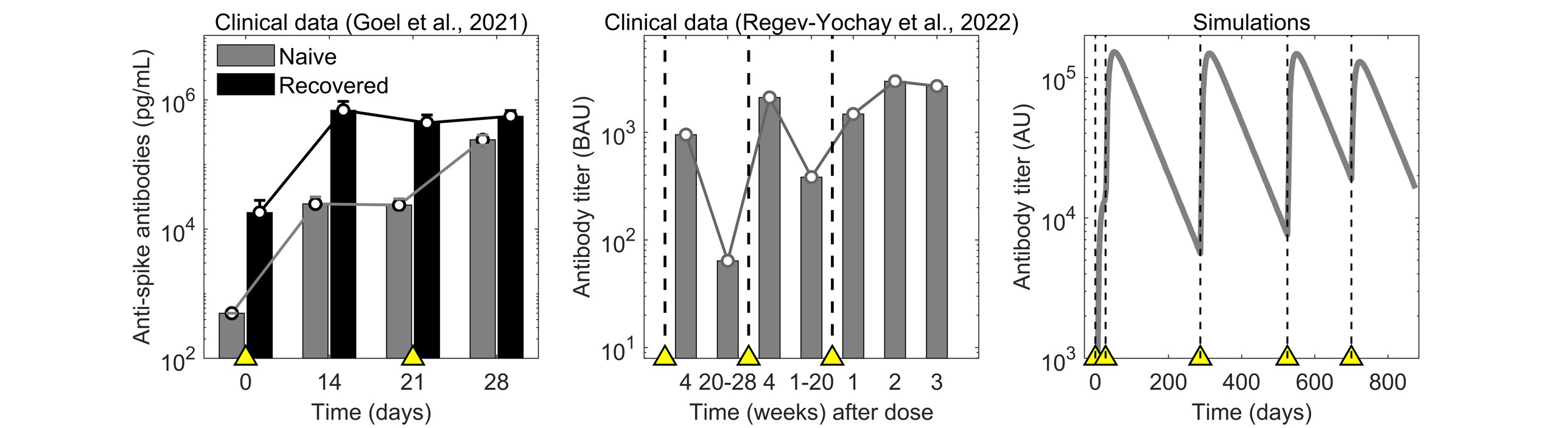

Supplement: Supplementary file 2 [file DataSheet_1.zip › Supplementary_Material_Codes/Fig2B.png]

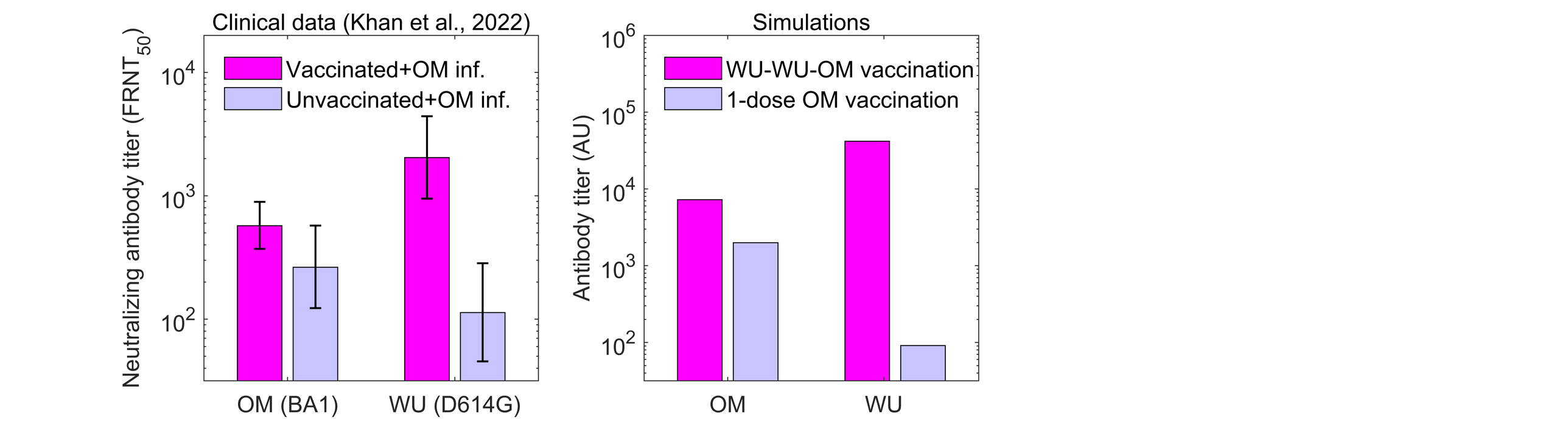

Supplement: Supplementary file 2 [file DataSheet_1.zip › Supplementary_Material_Codes/Fig2C.png]
